# Consumer Acceptance and Preference for Olive Oil Attributes—A Review

**DOI:** 10.3390/foods11233805

**Published:** 2022-11-25

**Authors:** Maria Elena Latino, Biagia De Devitiis, Angelo Corallo, Rosaria Viscecchia, Francesco Bimbo

**Affiliations:** 1 Department of Innovation Engineering, University of Salento, Campus Ecotekne, Via per Monteroni s.n., 73100 Lecce, Italy; 2 Department of Agricultural, Food, Natural Resources and Engineering Sciences (DAFNE), University of Foggia, Via Napoli, 25, 71122 Foggia, Italy

**Keywords:** olive oil, consumer behavior, consumer preference, sensory qualities, SEC, product attributes

## Abstract

Olive oil is largely produced in southern European countries. It encompasses a mix of search (e.g., price, color, packaging features), experience (e.g., taste), and credence attributes (e.g., organic, health claim). The importance of these attributes on consumers’ attitudes and preferences for Olive oil has been explored quite extensively in the past. However, a recent body of literature has focused on product nutritional information and health claims in shaping consumers’ attitudes and preferences for Olive oil. This work aims to offer an updated review of consumers’ acceptance and preferences for Olive oil features. Applying the Systematic Literature Review method, a sample of 47 studies published over the last 20 years was reviewed through descriptive and content analysis. The following attributes, grouped in search, experience, and credence categories, were discussed: origin, sustainability, brand, health and safety, the production process, packaging, color, taste and flavor, and product features. The discussion of marketing and research implications closes the study. The study provides an overview of the literature background of consumer behaviors of Olive Oil, investigating the recent literature focused on product nutritional information and health claims.

## 1. Introduction

Olive oil (OO) is a vegetable liquid fat obtained from olives (the fruit of Olea europaea). OO is largely produced in southern European countries such as Spain, Greece, and Italy, which together cover 70 to 75% of the worldwide OO production (EC, 2017). The European Union (Reg.EU 2568/91; Reg.EU 29/2012) and the International Olive Council (IOC) define the commercial quality grade of OO using analytical and organoleptic standards. Based on the results of chemical and sensory analyses, OO can be classified into three commercial categories: extra virgin olive oil (EVOO), virgin olive oil (VOO), or lampante olive oil. EVOO is the superior olive oil category with the highest quality and commercial value, while lampante is the lower one and is unsuitable for human use [1] (The Reg.EU 29/2012 classifies OO in three categories: “extra virgin olive oil” defined as the ‘superior category olive oil obtained directly from olives and solely by mechanical means’; then, “virgin olive oil” which is ‘olive oil obtained di-rectly from olives and solely by mechanical means’; lastly, “olive oil” composed of refined olive oils and virgin olive oils and it is ‘oil comprising exclusively olive oils that have undergone refining and oils obtained directly from olives’).

The olive oil (OO) sold in the marketplace encompasses a mix of search, experience, and credence attributes that impact consumer purchase decisions and the product’s perceived quality. Search attributes refer to visual attributes of the product (e.g., package size and material and the product’s color) for which consumers can seek pre-consumption information. Instead, experience attributes (e.g., taste and flavor) are attributes that are ascertained based on ex-post consumption of the product. Search and experience are prerequisite elements for consumers to discern the quality of OO [2].

Credence attributes, unlike search and experience attributes, are product quality characteristics that cannot be ascertained by direct experience (e.g., the place of origin, organic, health-related attributes and processing process techniques), thus consumers cannot know with certainty if such a “quality dimension” is actually within a product. Thus, credence attributes cannot be verified by consumers even after the purchase and consumption of the product [2,3]. Despite this, consumer studies pointed out that credence attributes have a positive impact on consumers’ attitudes toward food products and influence consumers’ preferences [4,5].

The importance of search, experience and credence attributes on consumers’ acceptance and preferences for OO was explored quite extensively as proved by Del Giudice et al. [6]. The authors collected and reviewed seventy-eight peer review studies and conference papers on consumers’ preferences for OO. The results overall show that consumers prefer EVOOs over VOO ones, a neutral taste rather than a pungent and bitter one, and EVOOs with credence attributes attached to the product. Researchers’ findings indicate that consumers prefer EVOO from countries or regional areas that are perceived to be strictly connected with the production of OO by consumers. Additionally, the organic label and producer’s brand strongly affect consumer preference, with the latter working as an additional guarantee of the information reported on the products.

A recent body of literature has focused on product nutritional information and health claims in shaping consumers’ acceptance and preferences for OO, which in some cases has a negative or little impact on consumers’ OO choices (see for instance [7,8,9,10,11]). Additionally, the study of consumer acceptance and preferences for OO with credence attributes related to the sustainability of the production process has gained increasing attention from scholars given the current global trend promoting sustainable production and consumption socio-economic model [3,12].

Thus, this work aims to offer an updated review of findings on consumers’ acceptance and preferences for OO attributes that include findings from recent studies on consumer and health-related attributes, as well as sustainable features. To this end, we selected relevant peer-reviewed studies published over the last 20 years on consumers’ acceptance and preferences for OO conducted in diverse contexts to be reviewed. Such a methodological approach allows us to gather relevant knowledge from a field where evidence is fragmented through a multi-step procedure to individuate and select studies [13]. Gaining more insight into consumers’ attitudes and preferences for a wide range of OO with several attributes may benefit both manufacturers and consumers, as will be illustrated throughout the manuscript. The manuscript is divided as follows: the next section describes the Materials and Methods of our study, Section 3 offers a descriptive review of the studies reviewed, while Section 4 contains an in-depth discussion of the 47 selected studies on consumer acceptance and preference for OO credence, search and experience attributes; lastly, Section 5 closes the manuscript with, respectively, the conclusion encompassing the marketing and research implications.

## 2. Materials and Methods

We systematically review the literature on consumer studies for OO in an attempt to critically analyze the body of knowledge [14] on product attributes that add value to olive oil products as highly accepted and preferred by consumers. In order to conduct a thorough literature review, a structured studies selection and analysis were performed in three main steps [14,15,16,17]: (1) question formulation; (2) defining the protocols for review; (3) analysis of the results and data synthesis. 

### 2.1. Question Formulation

Several authors suggest the selection of focused research questions to obtain more centered findings [18,19]. Starting from the evidence discussed in the Introduction Section, the following research questions were defined: “What are the search, experience and credence attributes able to affect the consumer acceptance and preferences for OO? To what extent consumer’s socio-economic and psychological factors shape the acceptance and preferences for OO?”

### 2.2. Definition of the Review Protocol

The review protocol included a keyword search selection to isolate the most relevant literature on the topic using Boolean operators (“AND” and “OR”) to increase the search accuracy [20]. Given the research focus, the following search scheme was defined to extract studies indexed on Scopus electronic scientific database (http://www.scopus.com accessed on 17 November 2022): (“food pattern” OR “food choice” OR “food preference” OR “consumer” OR “consumer consumption” OR “consumer preferences” OR “perception” OR “awareness” OR “understanding” OR “inference” OR “decision-making” OR “Preferences” OR “Willingness to Pay” OR “liking”) AND (“attributes” OR “attribute”) AND “olive oil”. Scopus database, managed by Elsevier, was chosen as the database to query because it represents a good quality literature source compared to other internet-based databases (e.g., Google Scholar) [20,21], and it is considered more inclusive than other databases such as Web of Science, which encloses only ISI-indexed journals [22]. The research, carried out in November 2020, was conducted according to the Scopus standard setting, searching in ‘Article title, Abstract, Keywords’ and providing an initial sample of 184 papers. The following selection criteria were applied to this sample:

- Exclude book chapters, conference papers and notes from the sample;
- Include English language studies in the sample;
- Include studies published in quality journals (Q1 and Q2 Scimago Journal quality rank) in the sample.

After applying the criteria listed above, we obtained a sample of 121 papers. 

The title and abstract of the studies that compose this sample were assessed to select only studies reporting knowledge about consumers’ attitudes and preferences in OO. After this screening, a final sample of 47 studies was identified and assessed through the content analysis of the full text. The quality-based choice of database and selection criteria was made in order to increase the quality of the results of this review.

### 2.3. Analysis of the Results and Data Synthesis

Descriptive and thematic analyses were conducted. Firstly, we applied a frequency analysis to provide an overview of the sample characteristics (e.g., methodology, sample size, and country of the study). Secondly, content analysis examines the body of knowledge in order to identify patterns in topics covered by the studies [23]. Content analysis can follow a qualitative or a quantitative approach [24]. In detail, qualitative content analysis was applied to evaluate the papers by discovering the meaning and organizing them into categories describing topics, themes and context [25]. The content analysis of the full text was realized through three steps: Firstly each study was assessed independently by each researcher who identified the aim and main results of the study. In the second stage, the individuals’ assessments were discussed collectively by a research group in order to identify a shared knowledge base.

**Figure 1:**
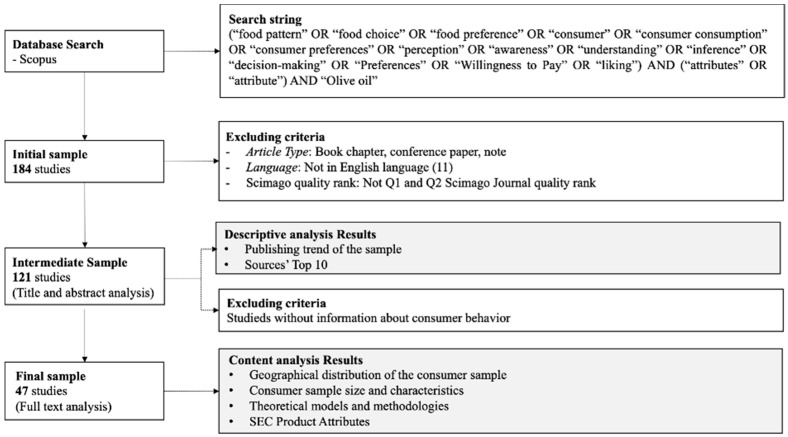
Research methodology.

The final sample was also analyzed in order to identify its descriptive characteristics such as: the geographical distribution of the consumers’ sample considered in the extant studies, the demographical characteristic of the consumers’ sample considered in the extant studies (e.g., age, gender, education, income, etc.), the theoretical models and methodologies used in the extant studies, and the product attributes analyzed in the extant studies. Figure 1 summarizes the proposed research methodology and the related findings.

## 3. Results and Discussion

In this section, the findings of the present study are presented and discussed.

### Descriptive Analysis

Mapping the body of knowledge through descriptive analysis is useful to identify trends, strengths and weaknesses of the extant studies [26].

**Figure 2:**
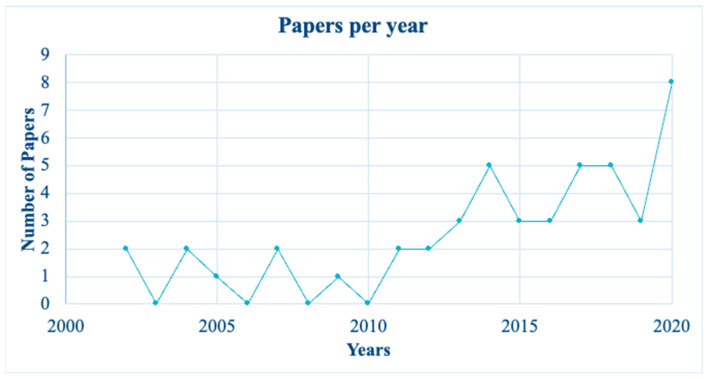
Publishing trend of the sample.

***Publishing trend.*** Figure 2 shows a trend in the number of papers published over time. The scientific panorama started to study the attributes of olive oil products in 2002. For the first ten years, the publication trend was substantially constant. Starting from 2011, there was an increase in the topic production, with a peak in 2020 denoting a high and increased interest in the OO food choice field.

**Figure 3:**
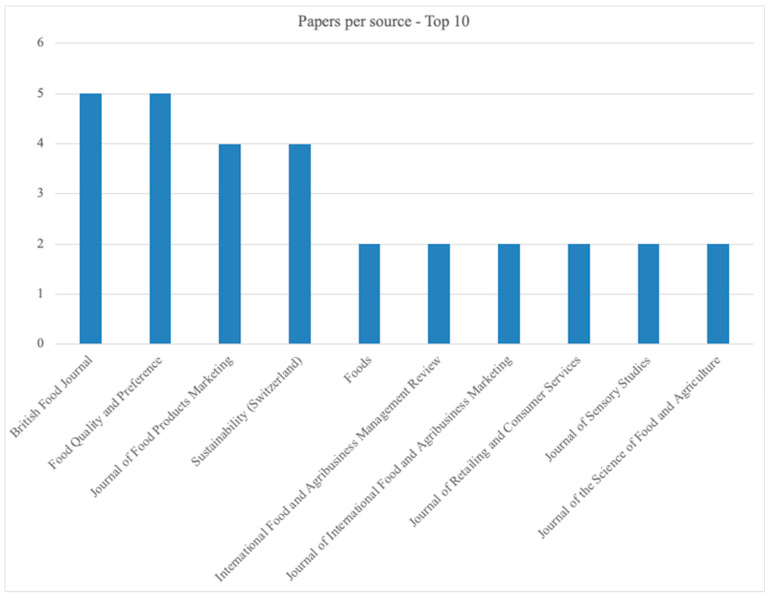
Source Top 10.

***Top 10 sources.*** Figure 3 shows the top 10 sources that encompass 63.8% of the papers of the sample. It is not surprising the presence of Journals focused on food and consumer fields.

## 4. Content Analysis

### 4.1. Study Characteristics

**Geographical distribution of the consumers’ sample in the extant studies.** Most of the studies included in this literature review focused on the OO preference for Italian (35.5%) [7,8,9,11,27,28,29,30,31,32,33,34,35,36,37,38,39] and Spanish (24.4%) [40,41,42,43,44,45,46,47,48,49,50] consumers, followed by studies focused on the OO preference of consumers from Greece (11.1%) [51,52,53,54,55], the USA (6.7%) [56,57,58], Chile [59,60], UK and German [61,62] and Tunisia [63,64] (4.4%), Japan [65], Brazil [66], Netherlands [30], Switzerland [67], Uruguay [68], France [63] (2.2%).

**Consumer size.** Each study, where indicated, was conducted on a consumer sample of different sizes. In order to provide a report, we group the size of the studies into four categories: less than 200 consumers, between 200 and 500 consumers, between 500 and 1000 consumers, and more than 1000 consumers. It emerges that 31.1% of the studies involve a sample with a size of less than 200 consumers [27,28,31,36,48,52,53,56,57,58,62,66,67,68], 33.3% of the studies involve a sample with a size between 200 and 500 consumers [30,36,39,40,42,43,44,45,46,49,59,60,63,64,69], 22.2% of the studies involve a sample with a size between 500 and 1000 consumers [29,32,33,37,41,47,51,61,70] and only 11.11% of the studies involve a sample with a size more than 1000 consumers [34,38,54,55,65]. In 8.8% of the reviewed studies, the sample size is not declared [7,8,9,50].

**Theoretical models and methodologies.** In the analysis sample, only five studies declared a well-established theory (e.g., Theory of Planned Behaviour, hedonic price model) [47,49,55,60,70]. Moreover, seven studies analyze a specific real olive oil product [27,28,50,56,60,67,70]. Among the several methodologies used to conduct the analyses, the following results are the most applied: choice experiment (22.2%) [7,31,34,39,40,41,49,59,65,69]; ANOVA (15.5%) [27,30,47,50,56,67,68]; conjoint analysis (13.3%) [8,35,42,43,53,64]; hedonic price model (6.6%) [9,29,70].

### 4.2. Product Attributes

#### 4.2.1. Credence Attributes

***Origin***. Among the credence attributes, the origin of the product was the most studied attribute. The product origin refers to the place where the production takes place. Thirty-two reviewed studies have focused on consumer acceptance and preferences for origin-related cues associated with the EVOO product. Most of the studies reported as origin cues were important drivers for EVOO or OO choices [7,8,9,11,27,30,32,34,36,39,41,43,44,45,46,49,51,53,57,59,60,61,63,65,66,67,69,71]. An exception to these findings was Mtimet et al.’s [64] study, according to which the region of origin attribute is not related to Tunisian consumers’ purchasing decisions of EVOO. Several studies pointed out as regional certifications are associated with consumers’ perceptions of olive oil authenticity, quality and healthiness, adding value to the product [30,31,51,59,60]. Additionally, other studies showed that the “origin of production” attribute was associated with a premium price in the Spanish market [41,43,46]. Spanish consumers consider elements related to the olive-oil quality both physical quality attributes (e.g., acidity, sensorial properties) and historical and identity values (e.g., origin, traditional production) [43]. Erraach et al. [43] found that Spanish consumers prefer the “PDO” label over the “country” of origin and “origin of production” information. Although the taste experience of the product may invert such preferences’ ranking, as found in Ballco and Gracia [41], where the “origin of production” was valued as more important than the “PDO” label after taste. According to Espejel et al. [44], consumers with low knowledge of OO bases their choice of consumption on intrinsic attributes (e.g., color, appearance, flavor, etc.), otherwise an expert consumer bases their choice of consumption on extrinsic attributes (e.g., origin). These results suggest the need to promote and increase the consumer knowledge of products protected under the PDO [45]. When the comparison of “origin of product” refers to a country of production, consumers from EVOO non-producing countries such as Japanese consumers prefer Italian EVOO over Spanish and Tunisian [65]. Additionally, Italian consumers prefer Italian EVOO rather than European [34] or Spanish [7] ones. However, Cicia et al. [31] and Scarpa and Del Giudice [69] suggested that Italian consumers are affected by home bias in purchasing EVOO as the domestic one was largely preferred over foreign EVOOs. These results were consistent with Tempesta and Vecchiato’s [39] findings that when investigating consumers’ preferences for Italian or Veneto EVOOs, suggest that Italian consumers largely prefer local products. Home bias was also detected in several studies testing the relevance of the production region among Italians and for which consumers are willing to pay a premium price ranging from +35% to +41.8% for 100% Italian EVOO [29,30,32,36]. Consistently, Greek consumers record home bias in their EVOO choices, preferring Greek olive oil over ones from other Mediterranean countries [53], as well as Chilean extra virgin olive oil consumers preferring domestic products over Italian and Spanish ones [59]. Home bias is less relevant in non-producing countries. For example, only 20% of Americans prefer local olive oil [57]. Comparing Netherlands and Italian consumers, Italians are more aware of the strict requirements needed to indicate the origin on the label [30]. Ilak Peršurić [61] showed that German consumers recognize a high value in origin attributes such as protected designations of geographical origin (PDO) and/or German original designation. Additionally, Roselli et al. [11] found that environmentally friendly consumers prefer domestic products to directly support rural landscape preservation. 

According to Jimenez-Guerrero et al. [66], the country of origin attribute is more capable of influencing inexperienced consumers than experienced consumers. From a sensory analysis point of view, it was noted that EVOO produced starting from olives produced in the same geographical area shared similar sensorial attributes [67].

***Sustainable-related attributes.*** The twenty-four studies reviewed have focused on consumer acceptance and preferences for sustainable-related attributes associated with the EVOO product. The studies reviewed pointed out that consumers highly accept organic EVOO and are also willing to pay a premium price to purchase a product produced according to sustainable farm practices. Specifically, the premium price associated with the “organic” attribute largely varies across studies ranging from +0.7 EUR/L [39] to +7.14 EUR/L [34]. The premium price of organic EVOO is driven by the consumers’ interest in lowering the environmental impacts of products consumed, as well as by the perception that organic EVOO is healthier compared to its conventional counterpart [30]. Indeed, organic EVOO is often defined as a “natural product”, “additive free”, “chemical residue free”, and “pure” [48,54]. Boncinelli et al. [7] found that consumers interested in EVOO with organic labels jointly selected health claims on it confirming an overlapping interest in a product’s health and sustainable attributes. Instead, the organic feature does not affect consumers’ organoleptic expectations and the overall quality perception [27,48]. Organic consumers were indicated as young with a medium–high education level and income. Women show marked preferences for organic EVOO compared to men [34,39,53]. Factors potentially hindering the consumer’s acceptance and preferences for organic EVOO were indicated as being the lack of knowledge about organic production methods; lack of knowledge and access to stores selling organic EVOOs; and lastly, the low level of trust in the organic information reported on the product’s label [47,48,54]. Those barriers may justify the results from two studies by Dekhili et al. [63] and Yangui et al. [49], which detected consumer disutility from purchasing organic EVOO, as well as consumers’ lack of interest in the organic attribute.

Although the majority of studies in the literature have focused on assessing consumer acceptance and preferences for the organic attribute, recently, scholars have explored consumer preferences for other sustainable attributes potentially available in olive oil such as those indicating that the product is “obtained from ancient trees”, or “produced in mountainous areas”, or “obtained with sustainable water use”, as well as whether the OO is produced from trees preserving the “traditional landscape” [34,39]. Those studies consistently found the existence of premium price associated with the product bearing such attributes and this premium ranges from 0.7 EUR/L for a product labeled as a “mountain product”, to 5.79 EUR/L for those products with olives collected “from ancient tree”. Thus, these findings pointed out that consumers are interested in the broader set of sustainable-related attributes potentially available in EVOO besides the organic ones and are willing to pay higher prices for such attributes [34,39].

***Brand.*** As a credence attribute, the brand was often considered to affect consumer acceptance and preferences for OO and EVOO in thirteen reviewed studies. Vlontzos and Duquenne [55] analyzed the Greek consumers’ WTP for several EVOO brands and found that around 20% of the consumers would accept paying more for a branded EVOO. Ballco and Gracia [41] studied the differences between processors’ leading brands and distributors’ private brands of Spanish EVOO, pointing out that leading brands are widely accepted among Spanish private labels to which consumers associated lower cost and quality. Antonialli et al. [66] pointed out that a price premium was associated with branded EVOO among Brazilian consumers. Contini et al. [32] and Espejel et al. [44] found that a brand shapes consumer acceptance and preference among consumers with a high “feeling”/knowledge of the product among Italian and Spanish consumers, respectively. Although, the average Italian’s acceptance and preference for EVOO are not affected by the product brand. Those results are consistent with findings from other studies, which found a lack of consumers attention to the EVOO brand among Italian [36], Greek [51] and Spanish [49] consumers, while [59] even recorded a negative influence in the final consumer price of a retailer brand in Chile.

***Safety and health-related statements.*** Six reviewed studies focused on consumer acceptance and preferences for safety and health-related statements associated with the EVOO product. One study review focused on Greeks’ preferences for EVOO with “HACCP” and “ISO” certifications, used as proxies for informing consumers about enhanced product safety features. Authors pointed out that consumers place a marginal interest in safety-related aspects taking it for granted in products available on the market, as well as recording a lower level of awareness for safety-related certification schemes [53].

All six studies cover consumer interest in and preference for health-related statements associated with EVOO products. Krystallis and Nees [53] pointed out that Greek consumers value health-related statements more than those indicating sustainable production practices (e.g., organic) when selecting EVOO products. Although, health-related statements recorded lower relevance when compared to information on the place of origin of the product. Greek consumers interested in health-related statements were young, educated, wealthy consumers of both genders with low average family size, approximately 2.8 members. Further, scholars in [54] pointed out that consumers perceive EVOO as the healthiest OO compared to other commercial OO categories surveying Greek consumers.

Comparable results from those obtained by Krystallis and Nees [53] were found by [32,36] when investigating Italian consumer preferences for EVOO products associated with a wide set of product attributes. The authors pointed out that the health-related statement is the second product attribute, after the one indicating the origin of the product, in orienting consumers’ preferences for EVOO. Boncinelli et al. [7] suggested that health-related statements play a marginal role in consumers’ EVOO choices. Such statements are of interest to half of the consumers surveyed, who would select an EVOO with health-related statements conditionally upon the product that already bears statements as organic and geographical indications. Instead, Cavallo et al. [9] found that a discounted price associated with an EVOO bearing a health-related statement suggests that consumers may find it difficult to process such information and for them to reject it. No attempt to segment consumers interested in health-related disclosure on the base of their social economics and psychographic characteristics were found in studies based in Italy.

Based on the literature reviewed, we conclude that the safety aspect of an EVOO product is taken for granted and that safety-related information is of no or limited interest to consumers selecting EVOO products. Instead, health-related statements are of interest, at least for a share of EVOO consumers, likely those consumers with a higher income or education as well as those with more of an interest in health. Related to the literature on health-related statements, it is worth mentioning that the heterogeneity of the findings available in the literature is large because authors have tested consumers’ preferences for a broad variety of health-related statements, for instance, terms such as “Best before”, “Keep until instructions”, “Additives free”, “Cholesterol free” [53], “Health claim” [7,36], “Substances beneficial for health” [32], and “Nutritional label” [9]. Additionally, result variations may depend on the sample used in the analysis, as three out of six cases were composed of less than 200 hundred observations collected in selected regions of Italy [9,36] and Greece [53]. Thus, findings may provide a partial picture of the impact of health-related statements on consumer preferences for EVOO.

**Production process**. Three reviewed studies have focused on consumer acceptance [30] and preferences [36,39] for statements associated with the OO production process such as “cold extraction” of EVOO and “handicraft milling technology”. Cavallo and Piqueras-Fiszman [30] first explored the impact of the “cold extraction” attribute on consumers’ perceived healthiness in a cross-sample of Dutch and Italian consumers, finding that this was not associated with a product’s higher healthiness perception and acceptance. Instead, more recent studies by Perito et al. [36] and Tempesta and Vecchiato [39] suggested that “cold extraction” or “handicraft milling technology” information is able to some extent to affect consumers’ preferences for EVOO. Perito et al. [36] found that “cold extraction” information scored as the fourth most relevant piece of information on the label for Italian consumers in selecting an EVOO product after the label’s region of production, sustainable production, and health claims information. Consumers’ interest in the production method (“cold extraction”) was of higher relevance for consumers who had higher knowledge of the quality attributes of EVOO and how production processes affect them. Lastly, Tempesta and Vecchiato [39] showed that “handicraft milling” information is as relevant as the organic production method as it is rated, in terms of interest, secondary solely to the place of EVOO production. This result likely depends on the fact that Italians consider EVOO obtained from the handicraft milling process as having a better taste compared to that obtained from the industrial extraction process, which standardizes the taste. Based on the studies reviewed in this section, we conclude that the production process information such as “handicraft milling” and “cold extraction” may play a role in the selection of EVOO products. However, their impact on EVOO consumers is underdeveloped and is not investigated in the large majority of studies on EVOO.

#### 4.2.2. Search and Experience Attributes

***Package features***. Fifteen reviewed studies have focused on consumer preferences for packaging attributes of the EVOOs. Italians and Spanish consumers pointed out that product package elements, such as brand, marginally affect consumer acceptance and preferences for EVOOs when compared with the product’s origin, quality certification [36], the type of oil, production system [42] and price [43]. A UK-based study has shown that price is the most important factor followed by package and its size in shaping consumer acceptance and preferences [62]. Additionally, Krystallis and Nees [53] found that company image and packaging attractiveness shape Greek consumer choice for extra virgin olive oil [53]. Furthermore, a “glass transparent bottle”d enhanced the perceived product quality image of the product among younger and well-educated consumers, as well as for the average consumer [53]. The latter prefer OO and EVOO in glass bottles than packaged in other materials (e.g., plastic, tin) that are sold at a lower retail price compared to products packaged in glass [41,59,62,70]. Although, OO and EVOO in a plastic bottle may be preferred due to the low risk of breakage compared to glass bottles during domestic use, as well as for inexpensive products purchased by low-income families [43]. Package size also shapes consumer acceptance and preferences for EVOO where consumers prefer to purchase products packaged in 1 L bottles and the larger package sizes are sold at lower prices regardless of whether consumers purchase them in a physical store or online [9,70]. Although consumers located in an EVOO-producing area that records high annual consumption of EVOO may prefer to purchase the product in bulk [59,64].

***Color, taste and flavor***. As pointed out in the twenty studies reviewed on the subject, sensory attributes may shape consumer acceptance and preferences according to whether the individual is a trained expert or an average consumer. The study by Delgado and Guinard [56] explored EVOO sensory analysis performed by trained experts who ranked those EVOOs with no organoleptic defects and with a fruity, bitter, and pungency taste of high quality. Several reviewed studies conducted in many countries found that the sensorial OO/EVOO features in which consumers place interest in terms of selecting the product are color, smell, taste, flavor, appearance and acidity. Specifically, most of the studies conducted in traditional producing and consuming countries have pointed out that sensory characteristics were important drivers in determining consumer preference and are attributes of high importance in the choice of EVOO [28,32,44,46,51,53], except for findings from a few studies [9,43,69], which found a marginal role in the EVOO’s sensory features (product’s appearance) when selecting a product among younger Italian and Spanish consumers. In the reviewed study, the taste is the main sensory feature shaping the preferences for EVOOs [44,51,53] over visual appearance, aroma and density [28].

However, consumers’ acceptance and preferences for EVOO color, taste, and flavor vary on whether the consumers are sampled in traditionally producing countries and non-traditional production ones. Studies found, in fact, that sensory attributes are more important for the former due to their higher level of knowledge about and familiarity with the product as well as its consumption [59,63]. Additionally, complex EVOO sensory profiles are highly appreciated by consumers who learned that product bitterness and pungency are associated with a higher content of chemicals beneficial for health. Instead, average consumers and those in non-traditional producing countries (e.g., Japan, Switzerland, Germany and the UK) strongly prefer EVOO with a sweet taste and reject bitter and pungent products [30,50,53,61,65,66,67,72]. A consumer with a higher level of knowledge about and familiarity with the product features, as well as those that consume EVOO regularly, are more likely to accept EVOO’s bitterness and pungency. This was consistently found among Italians (middle age, highly educated and mainly resident in northern Italy) [72] and Spanish consumers [50]. Cultural differences make Spanish consumers more likely to prefer EVOO with highly intense and pungent tastes as well as with a “fruity flavor”, “low degree of acidity” and “greenish-yellow color” [43,46,50]; Tunisian consumers prefer strong-flavored olive oils rather than neutral with “green colors” [64], as well as Uruguayans, who prefer EVOO with “green colors” [68].

## 5. Conclusions and Limitations

This study advanced the literature background about OO consumers’ attitudes and preferences through an updated literature review that considered the recent body of literature covering 20 years of studies. The studies reviewed pointed out that the average consumer prefers OO and EVOO sold at a low price with a greenish-yellow color, bottled in glass packaging of less than 1 L in volume rather than in large volumes or plastic containers. OO and EVOO with a neutral taste are preferred to the ones with a pungent bitter taste, on condition that the product is safe for human health. Additionally, products sold under well-known or local brands connected with an olive oil-producing area are, overall, preferred over others. Thus, producers may prefer to market EVOOs with a greenish-yellow color, plain taste, and packaged in a glass bottle. In addition, producers may want to first invest in developing and sustaining their brand image by connecting the latter with originality in OO/EVOO production as a means of increasing their revenues. 

Our findings also indicate that producers may benefit from differentiating their products, not only based on OO/EVOO taste, package feature, and brand, but also using credence attributes such as the “country of origin” (domestic product), “geographical indications”, as well as “sustainable” attributes (“organic”) that guarantee premium prices that are sizeable, in monetary terms, although this varies across the studies reviewed as well as the geographical settings considered. Related to the sustainable dimensions of the product, attributes such as OO/EVOO “obtained from ancient trees”, “produced in mountainous areas”, “obtained with sustainable water use”, produced from trees preserving the “traditional landscape” may further shape the consumer preferences for the product for the benefit of the producers, although these findings are limited and more research is needed. Instead, using health claims on EVOO to differentiate the product could be a winning strategy, given the literature’s contradictory evidence on consumer acceptance and preferences for health claims on EVOO.

From a methodological perspective, the widely used tool for assessing the use of product attributes or label information in the consumer decision-making process is several types of conjoint analysis. The wide use of such methodology generates findings that, to a large extent, depend on the other product attributes that were included in the conjoint design. Findings from conjoint-based studies point out that product attributes and label information can play a role in orienting consumer preference but that their role may be lower than documented. Indeed, conjoint methodology forces consumers to select fictional products generated using a limited set of product information by deviating away from everyday food shopping situations. There is no evidence about the actual consumers’ use of label information/product attributes in a real purchase situation. None of the reviewed studies were published using observation data at the point of purchase. Additionally, the studies reviewed in this work lack a theoretical framework or have employed rather general theoretical frameworks based on quality perception theories. 

First, from what was said above, there is the need to use the same methodology, over time, to achieve an understanding of whether consumers use label information/product attributes as well as whether a premium price exists for the many credence attributes potentially available for OO/EVOO. The differences in survey tools and sampling procedures employed in the available studies make it impossible to accurately assess to what extent consumers place interest in label information, including credence attributes, as well as the size of the premium price for the latter. Future research has to employ a constant set of product attributes, using large samples, to generate results with higher external validity. In addition, this approach would be useful to explore differences in consumer acceptance and preferences for OO features across multiple geographical contexts.

Secondly, there is a need for more micro-level studies on the consumer processes of attention, perception, and use of label information when presented with products in a shopping situation. This can be partially achieved using experimental lab studies involving eye-tracking methodology complemented by observational studies in real-world supermarkets. Thus, future works are encouraged to use observational studies set in food shops to capture granular information on consumer decision processes, such as the amount of attention consumers place on the food label and its elements, the overall label perception, the use of its information, and the effect of the complexity of the food environment on the label use.

Thirdly and lastly, there is a need for more research on the role of label information in actual decision-making settings since the dominance of conjoint designs in published studies forced respondents to look at a limited set of information on which to base their choices. Such tasks are more structured, as well as the product alternatives that are presented to the consumers being more comparable. Thus, there is the need to extend the research by employing studies at real points of purchase or in lab studies that reproduce supermarkets in terms of the complexity of the choice situation in order to generate findings that have a higher degree of external validity. Future studies are encouraged to use revealed preference data, such as scanner data, collected at the point of purchase. The use of this type of data will allow researchers to jointly assess the market value of multiple product attributes and infer the value consumers attach to them. Findings from studies using the stated preferences data will have a higher external validity, allowing for better result generalization.

## Data Availability

Not applicable.
